# Supplementary material for: Species-level bacterial community profiling of the healthy sinonasal microbiome using Pacific Biosciences sequencing of full-length 16S rRNA genes
Source: Microbiome. 2018 Oct 23;6:190. doi: 10.1186/s40168-018-0569-2 (PMC6199724; doi:10.1186/s40168-018-0569-2)
Supplement: Supplementary file 1 — Supplementary Text. (DOCX 56 kb) [file 40168_2018_569_MOESM1_ESM.docx]

**Additional file 1 –Supplementary Text**

**Species-level bacterial community profiling of the healthy sinonasal microbiome using Pacific Biosciences sequencing of full-length 16S rRNA genes**

Joshua P. Earl*, Nithin D. Adappa*, Jaroslaw Krol*, Archana S. Bhat, Sergey Balashov, Rachel L. Ehrlich, James N. Palmer, Alan D. Workman, Mariel Blasetti, Bhaswati Sen, Jocelyn Hammond, Noam A. Cohen, Garth D. Ehrlich**, Joshua Chang Mell**

* Contributed equally, ** Corresponding Authors

**Contents of Additional Files**

- **Additional File 1 – Supplementary Text** (this file)
  - Microbial Composition from Single Molecule Real Time sequencing (MCSMRT)
  - Sequence processing (pre-clustering)
  - Full-length 16S rRNA database for species-level classification
  - De novo OTU Clustering Pipeline
  - Identification of Amplicon Sequence Variants by Minimum Entropy Decomposition
  - Phylogenetic inference
  - QIIME2 OTU Clustering Pipeline
  - Mothur OTU Clustering Pipeline
  - Supplementary References
- **Additional File 2 – Supplementary Tables**
  - S1 Table: Expected composition of "Even" DNA Mock Community from BEI
  - S2 Table: Expected composition of DNA Mock Community from JGI-CAMI
  - S3 Table: PCR Primers Sequence
  - S4 Table: Filtering stats for mock communities
  - S5 Table: FL16S Denovo OTU Counts from QIIME2 with Greengenes v13_8 Classification
  - S6 Table: FL16S Closed OTU Counts from QIIME2 with Greengenes v13_8 Classification
  - S7 Table: BEI v3-v5 16S Denovo OTU Counts from QIIME2 with Greengenes v13_8 Classification
  - S8 Table: BEI v3v5 16S Closed OTU Counts from QIIME2 with Greengenes v13_8 Classification
  - S9 Table: OTU Counts and Taxonomic Classification for BEI Mock Community Classified with Mothur v1.35
  - S10 Table: OTU Count and Taxonomic Classification on Mock Community from Singer et. al.
  - S11 Table: Error Analysis of Reads Mapping to Positive Control E.coli MG1655 Reference
  - S12 Table: MCSMRT Classification of CAMI Mock Community
  - S13 Table: Classification Accuracies of Cami Community for Full-Length and V3-V5 Truncated 16S
  - S14 Table: Monophy test results from multi-species genera in the CAMI community
  - S15 Table: Sinonasal dataset with patient samples collected from sites as shown in Table 4.
  - S16 Table: Sinonasal Sample Statistics and NCBI BioSampleID
  - S17 Table: Sinonasal OTU Taxonomic Classification and Confidence
  - S18 Table: Read counts mapping to each OTU centroid for all sinonasal samples.
- **Additional file 3 – Supplementary Figures**
  - S1 Figure: Effect of primary filters on the number of reads
  - S2 Figure: Insert Size Distribution
  - S3 Figure. Effects of Host DNA on bacterial 16S yield
  - S4 Figure: Total CCS yield *vs.* PCR yields
  - S5 Figure: CCS reads mapping to the human genome from the sinonasal samples
  - S6 Figure: Primer matching filters
  - S7 Figure: Primer matching truncation and nucleotide variability against positive control *E. coli* forward and reverse primer matches
  - S8 Figure: Primer matching truncation and nucleotide variability against positive control *A. tumefacians* forward and reverse primer matches
  - S9 Figure: Histogram of the Number of Species per DB Cluster in NCBI.
  - S10 Figure: The effect of PCR cycle number and polymerase choice on OTU abundances in the BEI mock community
  - S11 Figure: The effect of PCR cycles and polymerase on abundances of chimeric molecules in the BEI mock community
  - S12 Figure: Substitution Errors in BEI Mock Community
  - S13 Figure: *E.coli* 16S copies and MED analysis
  - S14 Figure: Phylogenetic trees of well-resolved multi-species genera Clostridium and Desulfovibrio
  - S15 Figure: Phylogenetic trees of poorly-resolved multi-species genera Azotobacter and Nonlabens
  - S16 Figure: Phylogenetic trees of multi-species genera with improved species resolution using FL16S for Algoriphagus and Salegentibacter
  - S17 Figure: Phylogeny of Anaerococcus MED nodes from the sinonasal communities plus NCBI database entries
  - S18 Figure: Relationship between species-level confidence in centroid assignments and the number of species in the matching dbOTU
  - S19 Figure: Effective Number of Species as a function of sample read depth
- **Additional File 4** – Clustering of the NCBI 16S database into dbOTU
- **Additional File 5** – BEI phyloseq object
- **Additional File 6** – CAMI phyloseq object
- **Additional File 7** – Sinonasal physeq object

**Supplementary Methods**

Microbial Composition from Single Molecule Real Time sequencing (MCSMRT)

The MCSMRT pipeline is implemented using the programming language ruby and heavily relies on the bioruby project (<https://github.com/jpearl01/mcsmrt)>. Generation of CCS reads depends on SMRTportal v.2.3 and optionally SMRTpipe v.2.0. The remaining dependencies used were USEARCH (on drive5.com, USEARCH v8.1.1861_i86linux64 uparse pipeline, the default pipeline in Qiime1), BWA v0.7.10-r789, Sambamba v0.6.0, and MAFFT v7.273 (Edgar 2010, Li H. and Durbin R. 2009, A. Tarasov 2015, Kazutaka, and Standley. 2013).

Sequence processing (pre-clustering)

**Generation of demultiplexed CCS reads.** Reads of Insert Protocol (RoI) in the Pacific Biosciences software SMRTportal was used for demultiplexing. By default, we used a minimum of 5 CCS passes and a predicted accuracy score of 90 or greater. Reads were demultiplexed by their asymmetric barcode pairs (Table S3) into FASTQ files of circular consensus sequences (CCS) per barcode pair. An alternate method using only the command-line version of PacBio’s software SMRTpipe was developed for high-throughput applications. A detailed description on how to use of the ccs_smrt_pipe pipeline is on github, <https://github.com/rehrlich/ccs_smrt_pipe>. Using either demultiplexing method creates a directory of FASTQ files (one file for each pair of barcodes corresponding to each sample that was present in that PacBio cell). Each read in the FASTQ file is labelled with the number of CCS passes using ccs_passes.py (<https://github.com/PacificBiosciences/Bioinformatics-Training/raw/master/scripts/ccs_passes.py>) and the sample designation as part of the MCSMRT pipeline. All FASTQ files are then concatenated together.

**CCS read filtering and processing.** The principal filtration steps listed below were applied to the reads prior to their entry into the clustering pipeline:

***Sizing*** - The NCBI’s 16s Microbial Database does not contain reads smaller than 500 or larger than 2000 base pairs. Therefore, reads with lengths outside of these thresholds were removed.

***Host Mapping*** –To remove reads derived from off-target host DNA, reads were mapped to the GRCh37 human genome [(Zerbino et al. 2016)](https://paperpile.com/c/Gvg9L0/8aO6) using BWA v0.7.10-r789 (Li H. 2013) with default parameters. Unmapped reads were converted from BAM format back to FASTQ format for use in the rest of the pipeline.

***Primer mapping*** - Reads were aligned to the reference forward and reverse PCR primers, using search_oligodb in USEARCH [(Edgar 2010)](https://paperpile.com/c/Gvg9L0/QWsQ). Reads lacking a single forward and a single reverse primer were flagged (with maximum of 2 mismatches to reference; degenerate positions were accounted for). Artifacts of the demultiplexing process sometimes resulted in truncated primer sequences, which search_oligodb automatically fails to find. To account for this, reads with a single primer match were re-aligned with half the missing primer sequence, and matches were returned to the pool of reads used in the rest of the analysis.

***Primer trimming and orienting-*** The remaining reads were trimmed to remove the primer sequences using co-ordinates from the primer matching results. Because PacBio reads are not oriented with respect to strand, all reads were oriented 5`->3` by reverse complementation based on the primer match results.

**Taxonomic classification*-*** All primer-matched CCS reads were taxonomically labeled using the utax classifier described below, allowing for binning reads into clusters directly by taxonomic level (*e.g.* by genus), in addition to providing assignments for centroid OTU and MED node representatives.

Full-length 16S rRNA database for species-level classification

**Taxonomic labelling of NCBI 16S rRNA sequences*-*** Due to a dearth of species-level labels and/or full-length 16S sequences in popular 16S databases, we used the 16S Microbial database from the NCBI as the reference database for assigning taxonomy. This NCBI database contains 816 archaeal and 16,923 bacterial strains labeled as full-length 16S rRNA genes as of Oct 2015 and spanning 367 families and 2165 genera. The blast database was downloaded via an NCBI ftp (<http://ftp.ncbi.nih.gov/blast/db/>) and converted to FASTA using blastdbcmd from NCBI’s command line suite (blastdbcmd –db 16sMicrobial –out 16sMicrobial.fasta –outfmt %f –entry ‘all’). To convert the numeric headers of this database into a consistent taxonomic hierarchy, the file gi_taxid_nucl.dmp was used (<ftp://ftp.ncbi.nlm.nih.gov/pub/taxonomy/>). This dump file contains a list of all GI (GenInfo Identifier) numbers and their corresponding taxonomic IDs (txids) that map to full taxonomy of each microbial organism in the 16S rRNA Microbial Database. GI numbers were mapped to their txid, then the XML taxonomy database was parsed to obtain the corresponding full taxonomic lineage (using txid), which was added to that entry’s header and output in FASTA format. During this process, the headers were modified to conform to the required format for UTAX. In the final database, the headers were modified to only include the canonical designations of “Domain, Phylum, Class, Order, Family, Genus and Species”.

**Editing and filtering prior to utax classifier training-** Species name was concatenated with the genus name to avoid overlap of species names (as species names from different genera are not guaranteed to be unique, and this breaks the UTAX algorithm). Classifications that contained “sp, incertae, incerti, unknown, unclassified, chloroplast” were removed when training the UTAX confidence assignment model, as per the author’s recommendation using fastx_getseqs. These sequences were returned to the database after the model was trained. This allowed us to classify reads to the full database, but not confuse the model with broken or ambiguous taxonomic headers. Taxonomic confidences were trained with the train_utax command, and a binary database was created using the make_udbutax command.

**Other databases-** Additional databases were created that contained only the reference 16S sequences for the mock communities, as well as versions in which the full-length sequences were truncated based on primer-matching with primers that amplify distinct sets of variable regions (**Table S3, V1-V3, V3-V5**, and **V4** alone). The BEI reference database was created by downloading all whole genome sequences available via the Human Microbiome Project (HMP ftp://[public-ftp.hmpdacc.org/HMMC/](http://public-ftp.hmpdacc.org/HMMC/)).  Rnammer v1.2 [(Lagesen et al. 2007)](https://paperpile.com/c/Gvg9L0/ECSG) was used to predict the 16s loci; reads were labeled and added to a FASTA file.  The same procedure was done on the reference genomes for the CAMI community (<http://portal.nersc.gov/dna/metagenome/assembly/CAMI/reference/>), though this resulted in a database with many missing, fragmented, and incomplete 16S genes. The RDP gold database version microbiomeutil-r20110519 for use in chimera filtering was downloaded from <https://drive5.com/uchime/uchime_download.html> (Hass et. al. 2011). The Greengenes database v13_8 (99% OTUs) was downloaded from <https://data.qiime2.org/2018.6/common/gg-13-8-99-nb-classifier.qza>, and Silva database v132 (99% OTUs) was downloaded from <https://data.qiime2.org/2018.6/common/silva-132-99-nb-classifier.qza>, for use with classification in comparison pipelines in Qiime2 and Mothur (Mcdonald et al. 2012, Quast et al. 2013, Caporoso et al. 2010, Schloss et al. 2009).

*De novo* OTU Clustering Pipeline

**Expected error filtering –** Low quality reads were removed based on expected error (EE, by default EE >1 were removed) using fastq-filter (USEARCH) [(Edgar and Flyvbjerg 2015)](https://paperpile.com/c/Gvg9L0/yD58). An *in-silico* titration experiment with various expected error thresholds was performed to find the optimal EE level. Perfect classification of the BEI mock community at 3% sequence divergence level was achieved at an EE threshold of less than 1, therefore an expected error of 1 was used for all analyses, unless otherwise specified.

**Dereplication –** Reads were dereplicated via derep_fulllength (USEARCH), so that only unique sequences were kept, storing the count of identical reads in the header. The reads were sorted by descending order of abundance of each read sequence.

**Clustering into OTUs–** Reads were then clustered into OTUs using a 3% centroid-based divergence level via cluster_otus (USEARCH). Reads which differ by greater than 3% formed new OTU centroids and reads which differed by less than 3% were assigned to the closest matching OTU centroid. Reads with the highest dereplicated abundance form OTU centroids first.

**Identification of chimeras –** *CHIM1 filtering:* During clustering, each new centroid candidate read was checked against the current OTU centroid sequences and removed if alignment suggested the read was chimeric.*CHIM2 filtering:* Centroids were matched against the RDP gold database version microbiomeutil-r20110519 via uchime_ref (USEARCH) [(Hass et al. 2011, R. C. Edgar et al. 2011)](https://paperpile.com/c/Gvg9L0/kQbX) to identify chimeric centroids (CHIM2 chimeras). The NCBI 16S database provides a greater number of species (and especially species-level designations), however it has not been curated to avoid chimeric sequences. Since a chimera-free database was required, we used the curated RDP gold database.

**OTU Count Tables –** CCS reads output from the pre-clustering pipeline (by default, primer-matched but using no EE filter) were aligned to OTU centroids via usearch_global, assigning reads within a default 97% similarity threshold to existing OTUs. These results are then tabulated in to an OTU x counts matrix (**Additional File 5, 6, 7**).

Identification of Amplicon Sequence Variants by Minimum Entropy Decomposition

We found that Pacbio FL16S reads presented two specific challenges to using Amplicon Sequence Variant (ASV) analysis as implemented in DADA2 and MED, however we found a workaround with MED.

First, although both pipelines are reported to be robust to “true” indels, this is likely only applicable to short segments of 16S. FL16S alignments with diverse bacterial species are several times longer than any 16S gene, i.e. there are a large number of indel differences distinguishing diverse bacteria when including the full gene length. Both MED and DADA2 were unable to cope with this level of indel diversity. Thus, reads first needed to be clustered by similarity or taxonomic group, as in the original oligotyping method.

Second, the PacBio CCS error profile still has a much higher rate of indel errors than Illumina sequencing, albeit substantially improved over raw PacBio subread errors (Table 2.). Both ASV detection pipelines are currently unable to accommodate this type of error when using unaligned reads, since indel errors will then cause all downstream “alignment” differences to be read as true biological differences. Thus, after grouping reads by similarity (*i.e.* belonging to the same OTU) or taxonomic label, we aligned these sequences with MAFFT (einsi strategy). We were able to use MED when we then trimmed out alignment columns with a high percentage of gaps.

Thus upon OTU assignment or directly from taxonomic assignment, reads were parsed into separate FASTA files per cluster. These reads are then multiply aligned and used directly in the Minimum Entropy Decomposition (MED) pipeline. Our process was to take the subset of reads of interest, align them with MAFFT, trim columns from the alignments which consisted of >90% gap characters with trimal v1.2rev59, format headers to include each read’s sample first and specific read id second (separated by ‘|’ character), followed by the decompose command from MED in the Oligotyping pipeline v2.1 (using -t ‘|’ to define the header separating character and often requiring –skip-check-input flag) (Kazutaka, and Standley. 2013, Capella-Gutiérrez 2009, Eren et al. 2014).

Phylogenetic inference

Phylogenetic trees were built from subsets of primer-matched EE-filtered CCS reads (binned by OTU membership or direct read assignment to a genus).  Typically, MED decomposition of binned reads further reduced the final set for tree-building to the node representatives (along with the associated count of reads falling into that MED class). In most cases, additional sequences were added from the NCBI 16S database as references. Multiple alignments were produced with MAFFT v7.273 [(Katoh and Standley 2013)](https://paperpile.com/c/Gvg9L0/2FZE) using the ‘auto’ alignment parameter, and approximate maximum-likelihood tree were generated with FastTree v 2.1.10 (compiled using double precision to fix a problem with a lower-bound branch length downloaded from <http://www.microbesonline.org/fasttree/FastTreeDbl>; trees were built with the gtr model), using the default Shimodaira-Hasegawa test for node support (Price et al 2009, Price et al. 2010). For the highly abundant *Staphylococcus*-associated reads in the BEI mock community and human sinonasal communities, CCS reads were subsampled to those with >1 dereplicated count prior to combining with all NCBI sequences from *Staphylococcus* species prior to alignment and phylogeny inference. Trees were built using either full-length 16S reads or reads truncated to their V3-V5 region.

QIIME2 OTU Clustering Pipeline

QIIME2 clustering was performed following the standard online tutorials. In an attempt to fairly compare pipelines (and to accommodate specific artifacts of Pacbio sequences, i.e. unknown strand, primer matching) fastq reads that made it through the MCSMRT preclustering pipeline, and were subsequently EE filtered to <=1 were used. Fastq read data was imported using the ‘tools import’ command, reads were then dereplicated (using vsearch dereplicate-sequences) and filtered for chimeras (vsearch uchime-denovo, then feature-table filter-features, and feature-table filter-seqs). Reads were then either clustered denovo (vsearch cluster-features-de-novo) or clustered with closed OTU picking (vsearch cluster-features-closed-reference). Both FL16S, and truncated V3-V5 datasets were processed this way (**Table S5, S6, S7, S8**).

Mothur OTU Clustering Pipeline

Mothur v1.35 clustering was performed in a similar manner to (Schloss et al. 2016, Singer et al. 2016). In particular the steps taken were:

1. fastq_info (using pacbio=T)
2. unique_seqs
3. align.seqs (silva.nr_v132.align reference)
4. screen.seqs (to remove short or long aligned sequences)
5. filter.seqs (to remove columns with high gap characters)
6. unique.seqs (deduplicate any identitcal reads post-trimming)
7. pre.cluster (differences 2)
8. chimera.uchime
9. remove.seqs (remove chimeric reads)
10. dist.seqs (cutoff=.15)
11. classify.seqs(cutoff=80, trainset9_032012.pds.tax)
12. cluster (default)
13. summary.single(calc=sobs, label=0.03)
14. classify.otu

Commands relied on previous outputs in the Mothur pipeline, so these were performed in order as stated above. Analysis was performed on our BEI mock community data, after applying all levels of filtering up to and including the EE<=1 filter, to be comparing as similar a dataset as possible to our own clustering pipeline.

**Supplementary References:**

A. Tarasov, A. J. Vilella, E. Cuppen, I. J. Nijman, and P. Prins. Sambamba: fast processing of NGS alignment formats. Bioinformatics, 2015.

Capella-Gutiérrez, Salvador, José M. Silla-Martínez, and Toni Gabaldón. 2009. “trimAl: A Tool for Automated Alignment Trimming in Large-Scale Phylogenetic Analyses.” Bioinformatics 25 (15): 1972–73.

Caporaso, J. Gregory, Justin Kuczynski, Jesse Stombaugh, Kyle Bittinger, Frederic D. Bushman, Elizabeth K. Costello, Noah Fierer, et al. 2010. “QIIME Allows Analysis of High-Throughput Community Sequencing Data.” Nature Methods 7 (5): 335–36.

[Charif, Delphine, and Jean R. Lobry. 2007. “SeqinR 1.0-2: A Contributed Package to the R Project for Statistical Computing Devoted to Biological Sequences Retrieval and Analysis.” In *Biological and Medical Physics, Biomedical Engineering*, 207–32.](http://paperpile.com/b/Gvg9L0/POry)

[Cole, James R., Qiong Wang, Jordan A. Fish, Benli Chai, Donna M. McGarrell, Yanni Sun, C. Titus Brown, Andrea Porras-Alfaro, Cheryl R. Kuske, and James M. Tiedje. 2014. “Ribosomal Database Project: Data and Tools for High Throughput rRNA Analysis.” *Nucleic Acids Research* 42 (Database issue): D633–42.](http://paperpile.com/b/Gvg9L0/t4sf)

[Edgar, R. C., B. J. Haas, J. C. Clemente, C. Quince, and R. Knight. 2011. “UCHIME Improves Sensitivity and Speed of Chimera Detection.” *Bioinformatics*  27 (16): 2194–2200.](http://paperpile.com/b/Gvg9L0/kQbX)

[Edgar, Robert C. 2010. “Search and Clustering Orders of Magnitude Faster than BLAST.” *Bioinformatics*  26 (19): 2460–61.](http://paperpile.com/b/Gvg9L0/QWsQ)

[Edgar, Robert C., and Henrik Flyvbjerg. 2015. “Error Filtering, Pair Assembly and Error Correction for next-Generation Sequencing Reads.” *Bioinformatics*  31 (21): 3476–82.](http://paperpile.com/b/Gvg9L0/yD58)

Eren, A. Murat, Hilary G. Morrison, Pamela J. Lescault, Julie Reveillaud, Joseph H. Vineis, and Mitchell L. Sogin. 2015. “Minimum Entropy Decomposition: Unsupervised Oligotyping for Sensitive Partitioning of High-Throughput Marker Gene Sequences.” The ISME Journal 9 (4): 968–79.

Haas, Brian J., Dirk Gevers, Ashlee M. Earl, Mike Feldgarden, Doyle V. Ward, Georgia Giannoukos, Dawn Ciulla, et al. 2011. “Chimeric 16S rRNA Sequence Formation and Detection in Sanger and 454-Pyrosequenced PCR Amplicons.” Genome Research 21 (3): 494–504.

[Katoh, Kazutaka, and Daron M. Standley. 2013. “MAFFT Multiple Sequence Alignment Software Version 7: Improvements in Performance and Usability.” *Molecular Biology and Evolution* 30 (4): 772–80.](http://paperpile.com/b/Gvg9L0/2FZE)

[Lagesen, K., P. Hallin, E. A. Rodland, H-H Staerfeldt, T. Rognes, and D. W. Ussery. 2007. “RNAmmer: Consistent and Rapid Annotation of Ribosomal RNA Genes.” *Nucleic Acids Research* 35 (9): 3100–3108.](http://paperpile.com/b/Gvg9L0/ECSG)

Li H. and Durbin R. (2009) Fast and accurate short read alignment with Burrows-Wheeler Transform. Bioinformatics, 25:1754-60. [PMID: 19451168]

Li H, 2013. Aligning sequence reads, clone sequences and assembly contigs with BWA-MEM. [rXiv:130a3.3997v2](http://arxiv.org/abs/1303.3997)

McDonald, Daniel, Morgan N. Price, Julia Goodrich, Eric P. Nawrocki, Todd Z. DeSantis, Alexander Probst, Gary L. Andersen, Rob Knight, and Philip Hugenholtz. 2012. “An Improved Greengenes Taxonomy with Explicit Ranks for Ecological and Evolutionary Analyses of Bacteria and Archaea.” The ISME Journal 6 (3): 610–18.

Price, Morgan N., Paramvir S. Dehal, and Adam P. Arkin. 2009. “FastTree: Computing Large Minimum Evolution Trees with Profiles instead of a Distance Matrix.” Molecular Biology and Evolution 26 (7): 1641–50.

[Price, Morgan N., Paramvir S. Dehal, and Adam P. Arkin. 2010. “FastTree 2 – Approximately Maximum-Likelihood Trees for Large Alignments.” *PloS One* 5 (3): e9490.](http://paperpile.com/b/Gvg9L0/CacN)

Schloss, Patrick D., Sarah L. Westcott, Thomas Ryabin, Justine R. Hall, Martin Hartmann, Emily B. Hollister, Ryan A. Lesniewski, et al. 2009. “Introducing Mothur: Open-Source, Platform-Independent, Community-Supported Software for Describing and Comparing Microbial Communities.” Applied and Environmental Microbiology 75 (23): 7537–41.

Schloss, P. D., M. L. Jenior, C. C. Koumpouras, S. L. Westcott, and S. K. Highlander. 2016. “Sequencing 16S rRNA Gene Fragments Using the PacBio SMRT DNA Sequencing System.” *PeerJ* 4: e1869.

Singer, E., B. Bushnell, D. Coleman-Derr, B. Bowman, R. M. Bowers, A. Levy, E. A. Gies, et al. 2016. “High-Resolution Phylogenetic Microbial Community Profiling.” *The ISME Journal* 10 (8): 2020–32.

Quast, Christian, Elmar Pruesse, Pelin Yilmaz, Jan Gerken, Timmy Schweer, Pablo Yarza, Jörg Peplies, and Frank Oliver Glöckner. 2013. “The SILVA Ribosomal RNA Gene Database Project: Improved Data Processing and Web-Based Tools.” Nucleic Acids Research 41 (Database issue): D590–96.

[Zerbino, Daniel R., Nathan Johnson, Thomas Juetteman, Dan Sheppard, Steven P. Wilder, Ilias Lavidas, Michael Nuhn, et al. 2016. “Ensembl Regulation Resources.” *Database: The Journal of Biological Databases and Curation* 2016 (February). doi:](http://paperpile.com/b/Gvg9L0/8aO6)[10.1093/database/bav119](http://dx.doi.org/10.1093/database/bav119)[.](http://paperpile.com/b/Gvg9L0/8aO6)
