# Supplementary material for: Species-level bacterial community profiling of the healthy sinonasal microbiome using Pacific Biosciences sequencing of full-length 16S rRNA genes
Source: Microbiome. 2018 Oct 23;6:190. doi: 10.1186/s40168-018-0569-2 (PMC6199724; doi:10.1186/s40168-018-0569-2)

Additional File 3 –Supplementary Figures

Species-level bacterial community profiling of the healthy sinonasal microbiome using Pacific Biosciences sequencing of full-length 16S rRNA genes

Joshua P. Earl*, Nithin D. Adappa*, Jaroslaw Krol*, Archana S. Bhat, Sergey Balashov, Rachel L. Ehrlich, James N. Palmer, Alan D. Workman, Mariel Blasetti, Bhaswati Sen, Jocelyn Hammond, Noam A. Cohen, Garth D. Ehrlich**, Joshua Chang Mell**

* Contributed equally, ** Corresponding Authors

S1 Figure. **Effect of primary filters on the number of reads.** The Venn diagrams on the left depict the number of reads that pass each filter and the ones on the right depict the number of reads that did not. The three sets represent the reads in (A) BEI, (B) CAMI, and (C) sinonasal communities.

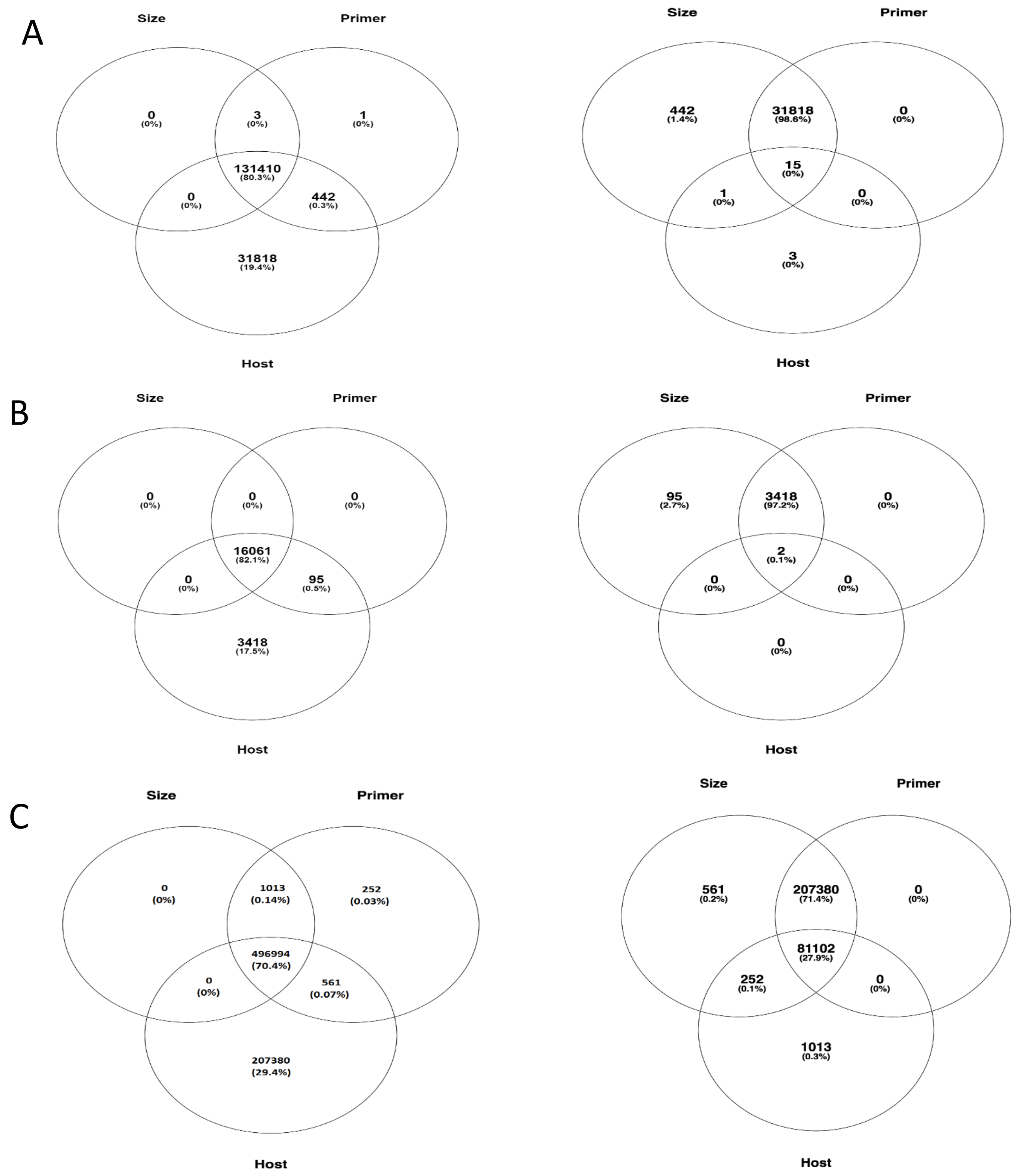

S2 Figure. Insert size distribution. The x-axis shows the length of the Circular Consensus Sequence (CCS) reads. The y-axis is the percentage of reads that contains a base at that position.

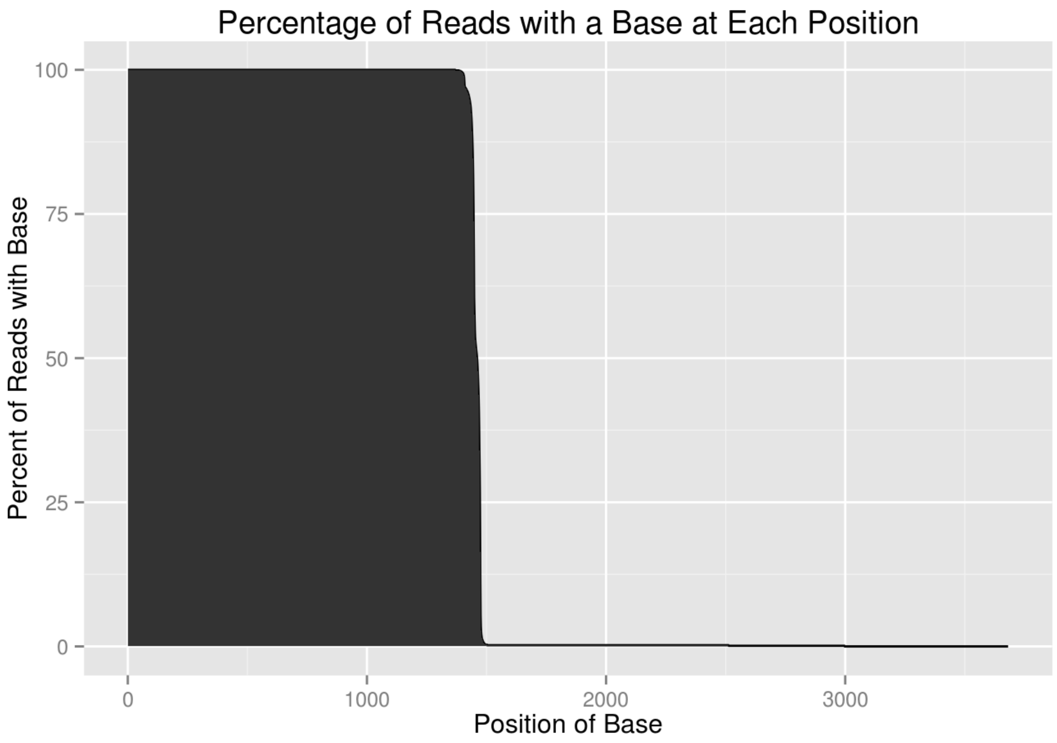

S3 Figure. Effects of Host DNA on bacterial 16S yield. (A) Total yield of CCS reads in BEI mock community samples run with two distinct polymerases (accu = AccuPrime, gotaq = GoTaq), two PCR cycle numbers (22 or 35), and with or without 10-fold excess by mass of human DNA extracted from human lymphoblast lung cells (*U937*) (40 ng added). (B) Percent of size-filtered CCS reads (log-scaled) that map to the human hg38 reference sequence in the sinonasal microbiome samples. Color indicates patient ID (indicated by legend). Letter code indicates site ID, as indicated in **Figure 6**. (C) Post-human filtered CCS reads.

A.

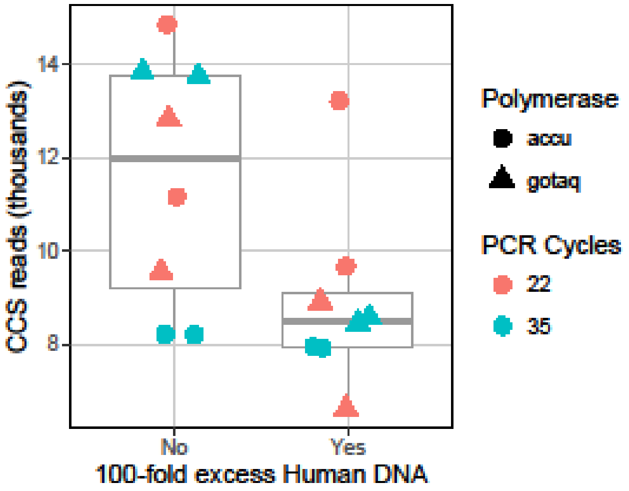

B.

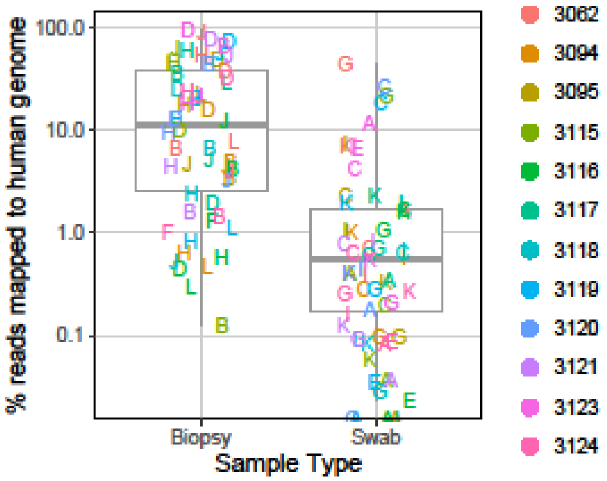

C.

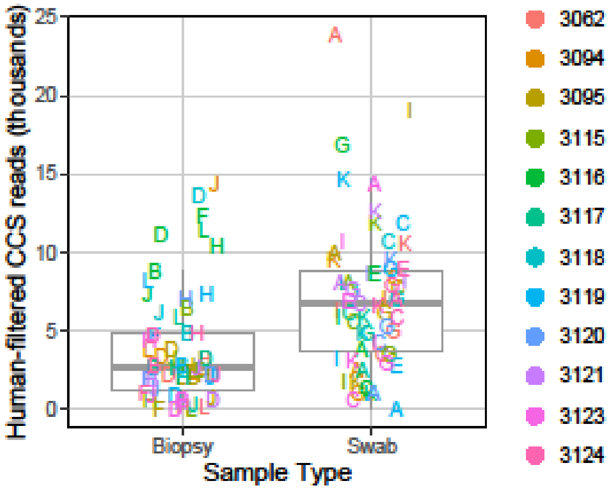

S4 Figure. Total CCS yield *vs.* DNA yield *vs.* PCR yields. (A) Scatter plot PCR concentration (ng/µl) and CCS yield. Color of the dots represents sample type (swab/biopsy) and shape of the dots represents the site ID pairs as per **Figure 6**. (B) Scatter plot of PCR concentration (ng/µl) and DNA concentration (ng/µl). Color of the dots represents sample type (swab/biopsy) and shape of the dots represents the site ID pairs as per **Figure 6**.

A.

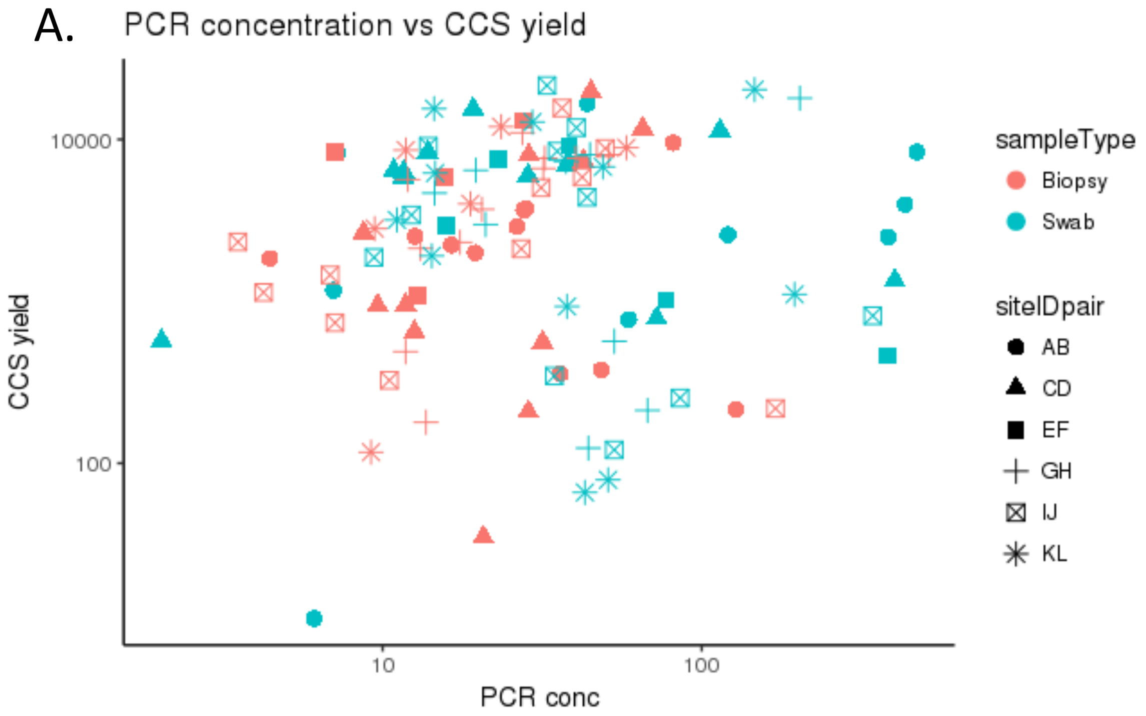

B.

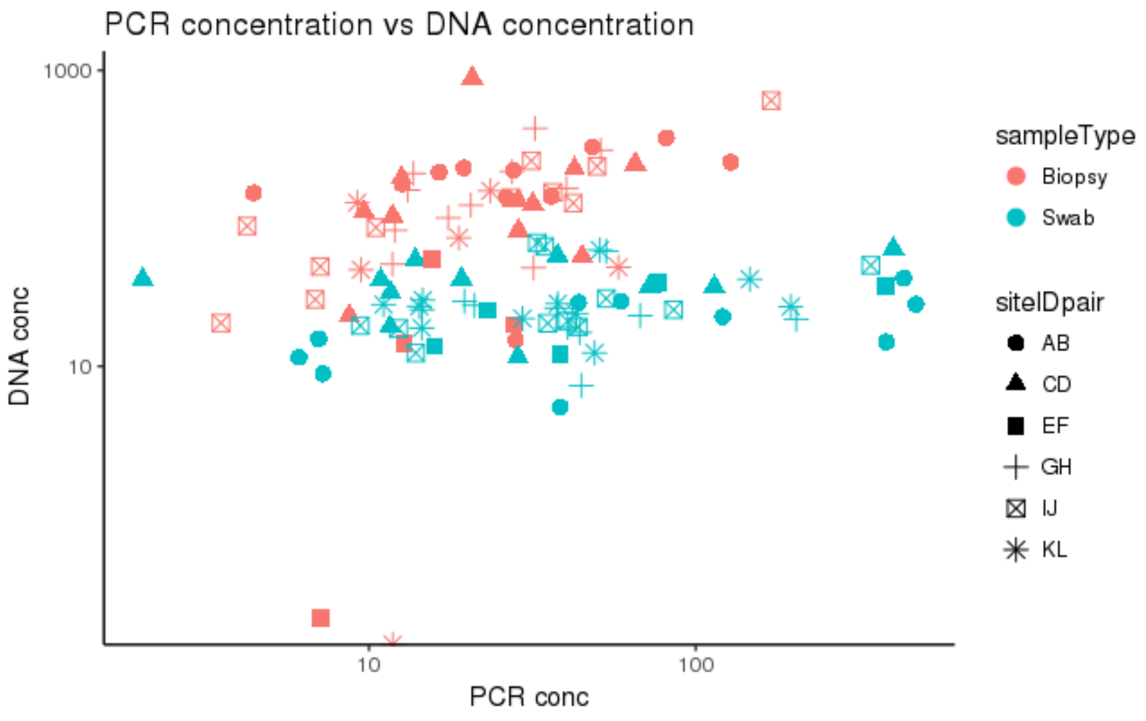

S5 Figure. CCS reads mapping to the human genome from the sinonasal samples. (A) Frequency spectrum showing the number of reads *vs* number of mapped read start positions. (B) Read counts across the human genome (hg19). Facets represent the chromosomes in the human genome, x-axis marks the position within each chromosome, and y-axis contains the number of reads that map to the different chromosomes.

A.

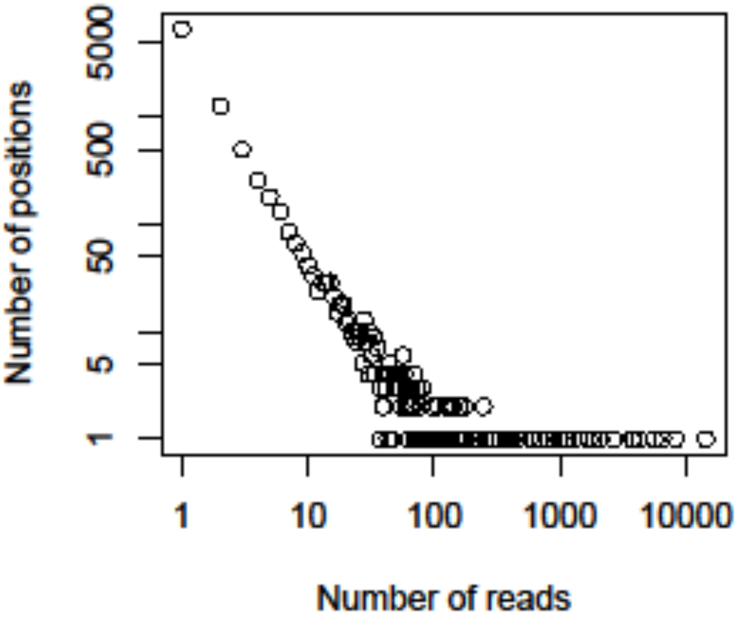

B.

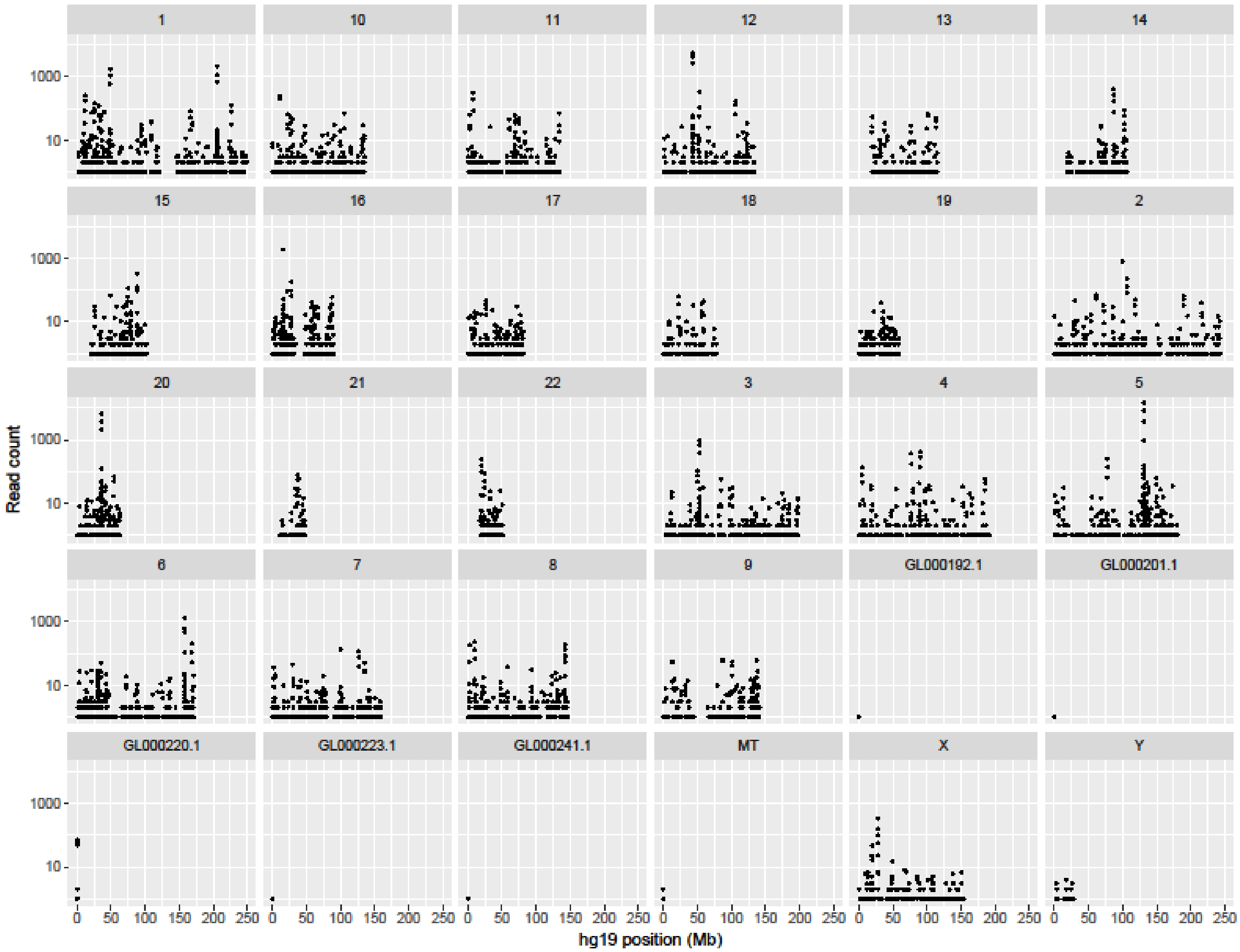

S6 Figure. Primer matching filters. Representation of the effect of primer matching on the number of reads. The x-axis marks the types of primer matching that can occur and the y-axis represents the number of reads (in thousands). The colors represent if both the primers passed or not.

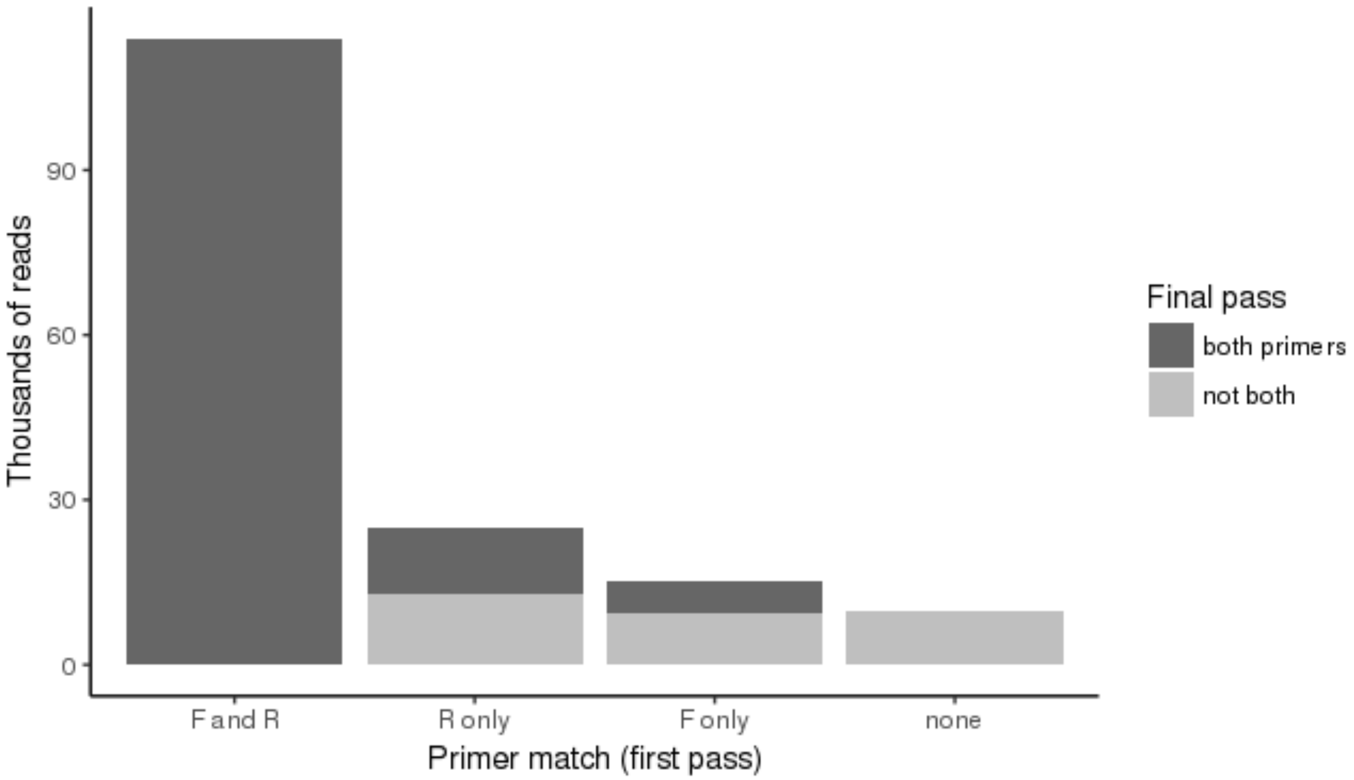

Figure S7. Primer matching truncation and nucleotide variability against positive control *E.coli* forward and reverse primer matches. (A) Barplot to show the truncation of forward primer in reads belonging to *Escherichia coli* positive controls. The x-axis has each position within the primer region of the reads and the y-axis shows the frequency of gaps. The sequence logo shows non-specific primers based on the degenerate nucleotides. (B) Barplot to show the truncation of reverse primer in reads belonging to *Escherichia coli* positive controls. The x-axis has each position within the primer region of the reads and the y-axis shows the frequency of gaps. The sequence logo shows non-specific primers based on the degenerate nucleotides.

A.

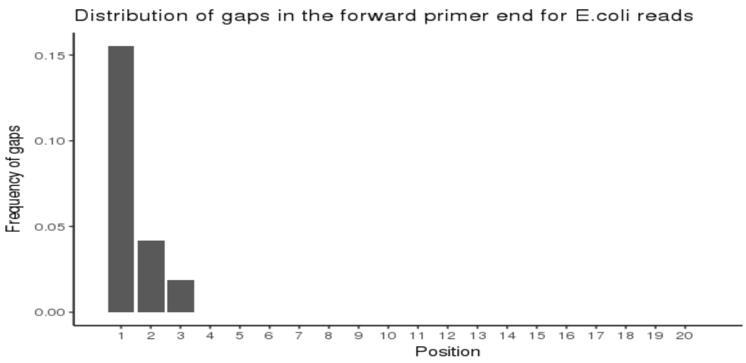

B.

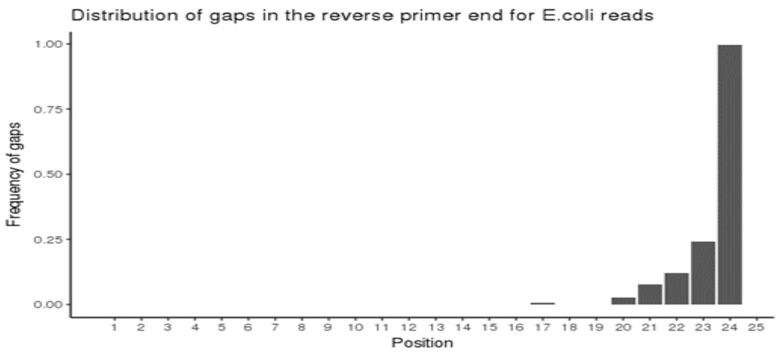

True Sequence GAAGAGTTTGATCATGGCTCAG

Primer Sequence GRAGRGTTYGATYMTGGCTCAG

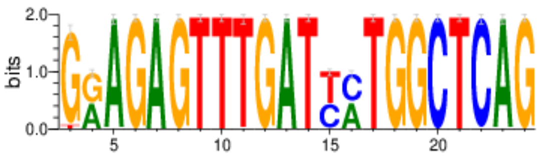

True Sequence

Primer Sequence AAGTCGTAACAAGGTARCYGTA
AAGTCGTAACAAGGTAACCGTA

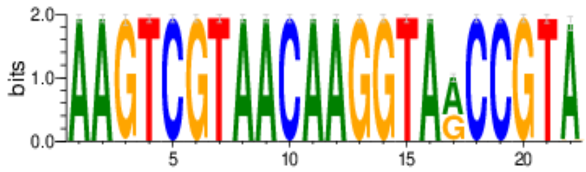

Figure S8. Primer matching truncation and nucleotide variability against positive control *A.tumefaciens* forward and reverse primer matches. (A) Barplot showing the forward primer sequences from reads belonging to *Agrobacterium tumefaciens* positive controls. The x-axis has each position within the primer region of the reads and the y-axis shows the frequency of gaps. The sequence logo shows non-specific primers based on the degenerate nucleotides. (B) Barplot showing the reverse primer sequences from reads belonging to *Agrobacterium tumefaciens* positive controls. The x-axis has each position within the primer region of the reads and the y-axis shows the frequency of gaps. The sequence logo shows non-specific primers based on the degenerate nucleotides.

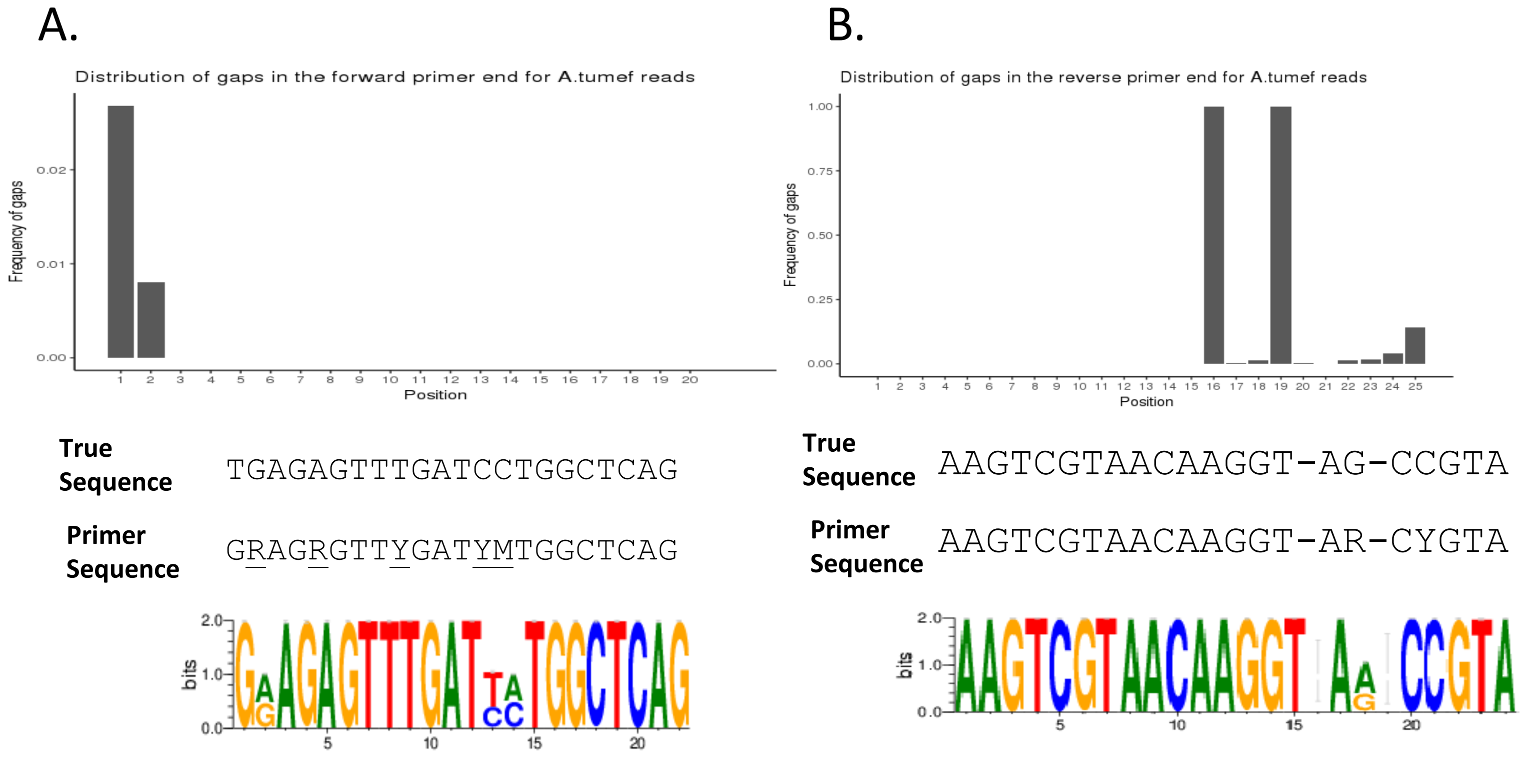

S9 Figure. Histogram of the Number of Species per DB Cluster in NCBI. Histogram of the number of species per cluster in NCBI. Clusters in NCBI were obtained by average-linkage hierarchical clustering of all reads in the NCBI database with a 3% identity cutoff. The y-axis is the number of clusters observed, and the x-axis is the number of species in those clusters.

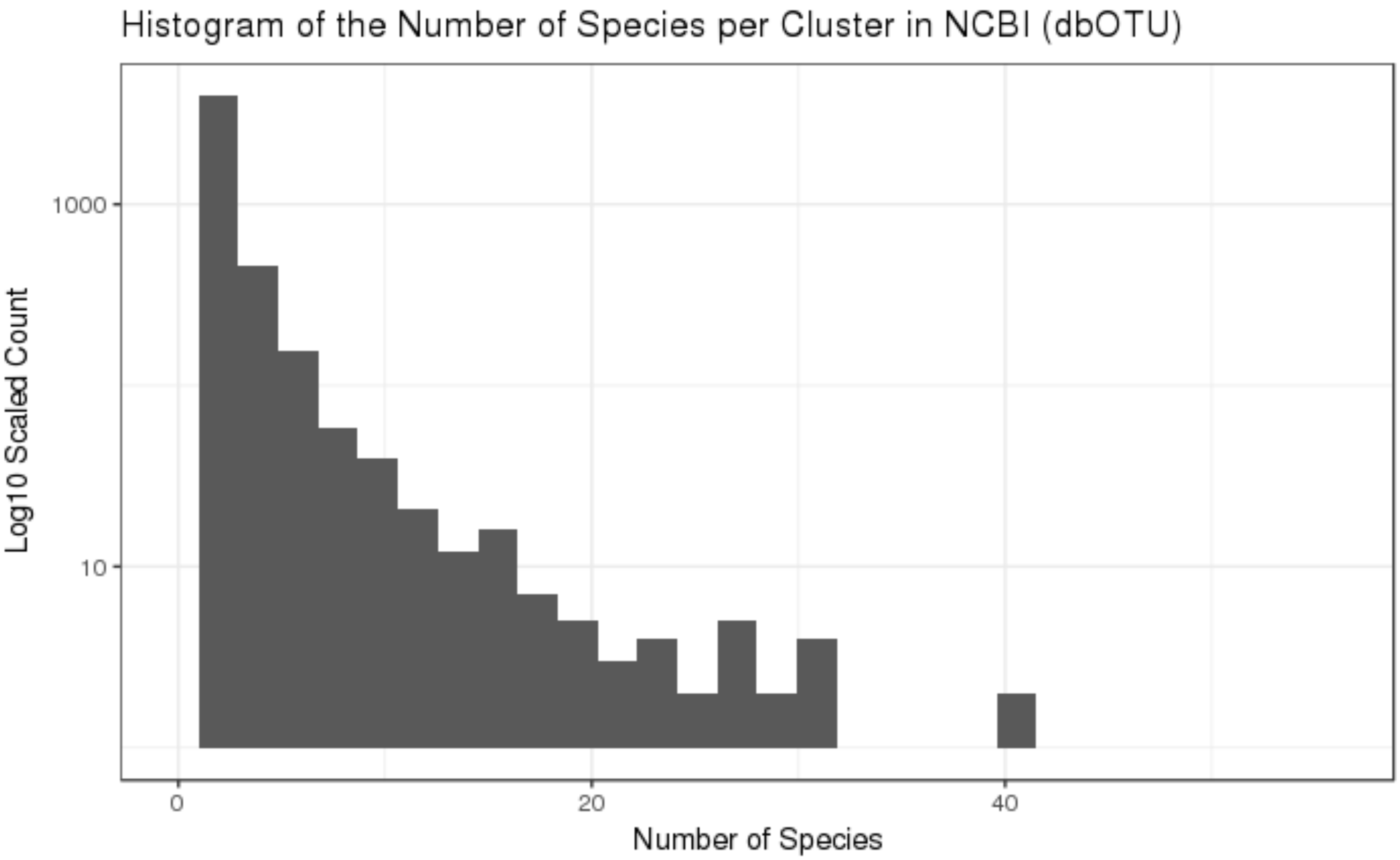

S10 Figure. The effect of PCR cycle number and polymerase choice on OTU abundances in the BEI mock community. OTUs ranked by relative abundances for each species detected in the BEI mock community under 8 separate reactions and library preps, using either GoTaq or AccuPrime polymerases, either 22 or 35 PCR cycles, and with or without 10-fold excess human DNA (U937 human cell line lymphoblast lung) by mass. Species abbreviations as in Table S1.

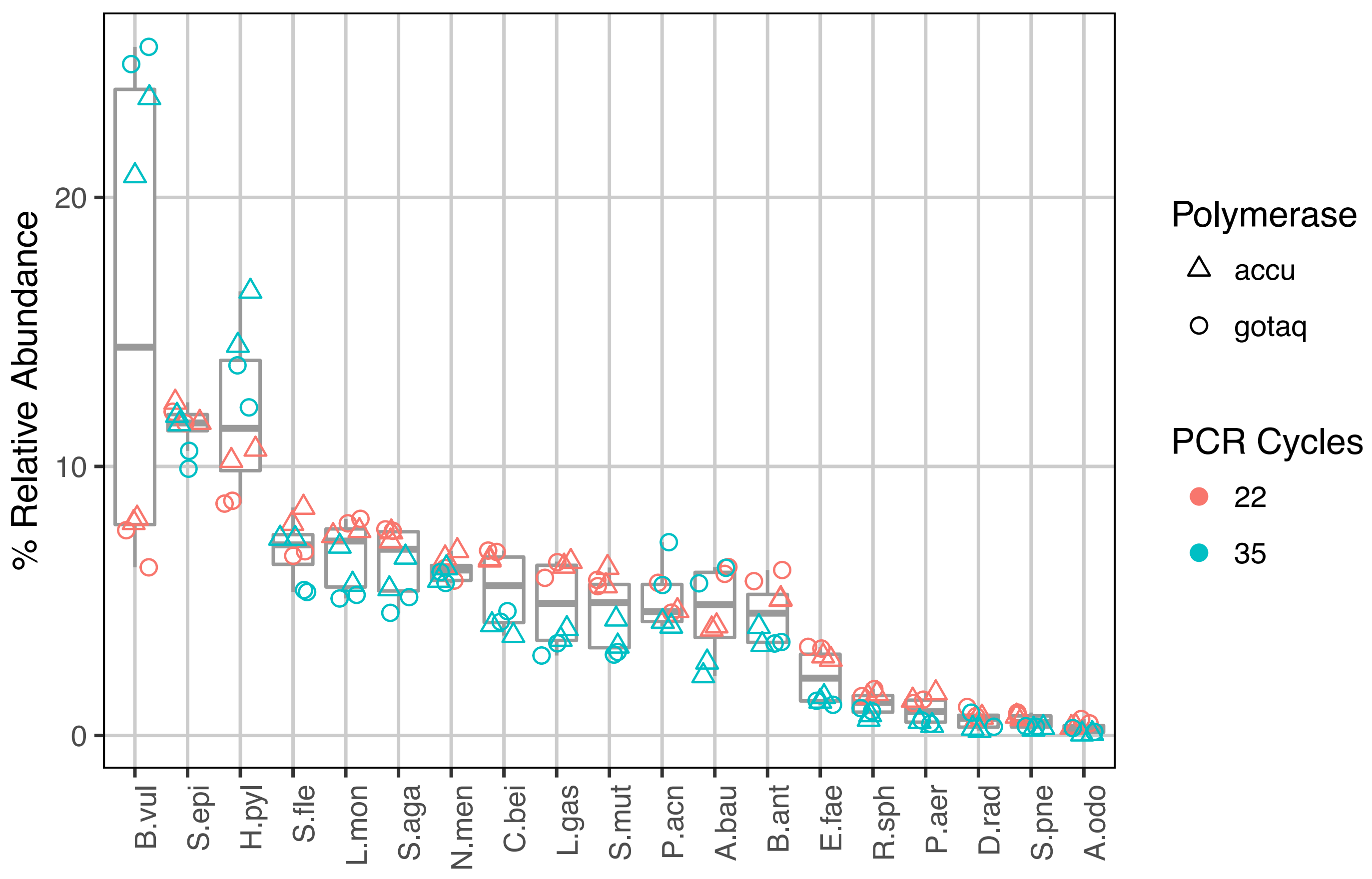

S11 Figure. The effect of PCR cycles and polymerase on abundances of chimeric molecules in the BEI mock community. Each primer-matched read was aligned to the reference 16S rRNA gene sequences from BEI with ublast, and the best hit was taken as the source sequence. Identification of CHIM1 chimeras among all reads was determined by OTU clustering with no EE filter. The barplot shows the total read counts seen for each PCR condition divided into chimeric and non-chimeric counts.

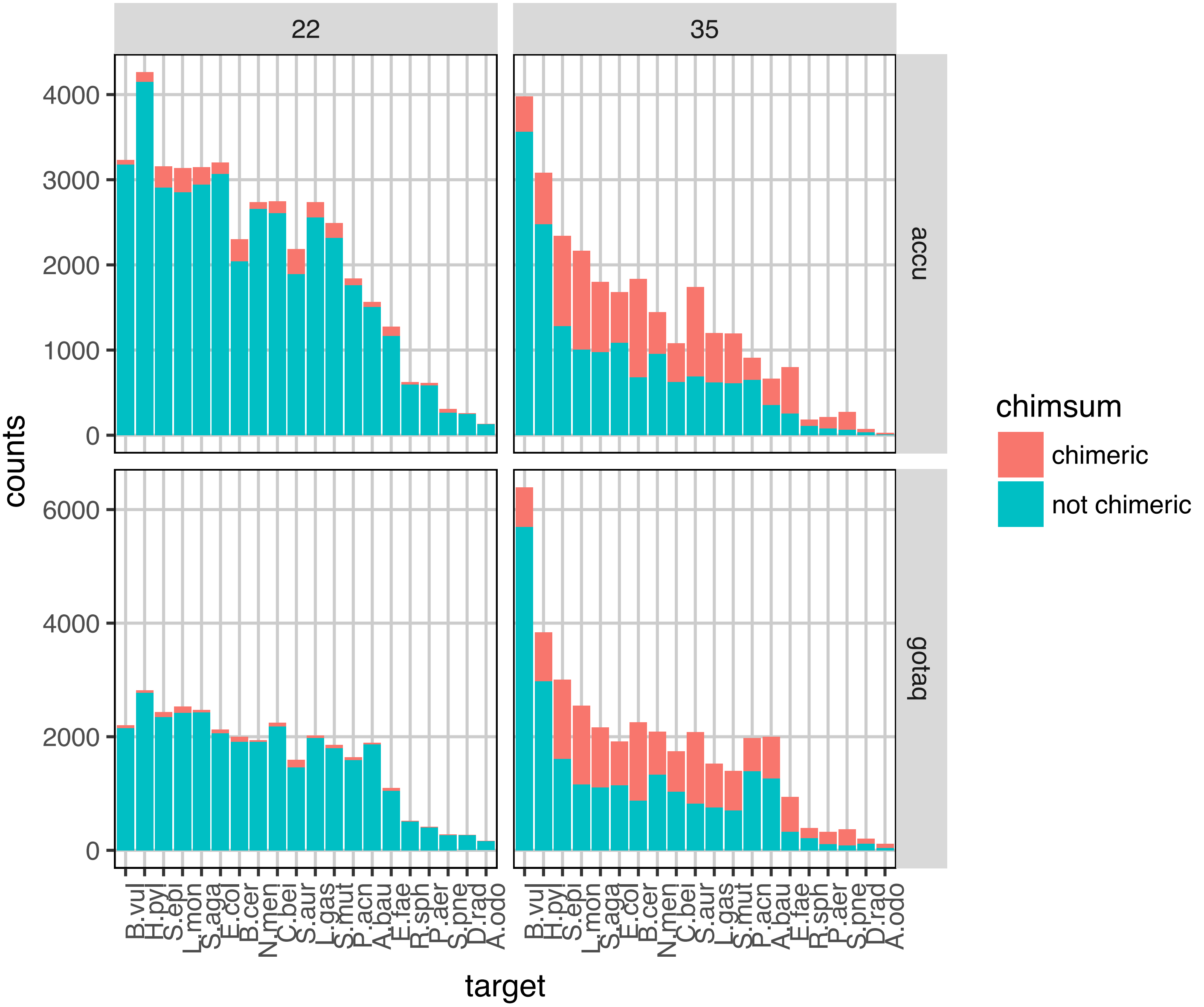

S12 Figure. Substitution Errors in BEI Mock Community. Plots were generated from reads without an EE filter. (A) Boxplot of the number of mismatches (not including gaps) to the closest reference sequence for each read, binned by likely source organism and split by the polymerase used (AccuPrime or Gotaq). Upper bound of 20 mismatches. (Mean mismatch Accuprime 12.9, Gotaq 15.2) (B) Histogram of the number of mismatches observed in each read. (C) Histogram of the number of sequences with expected error value.

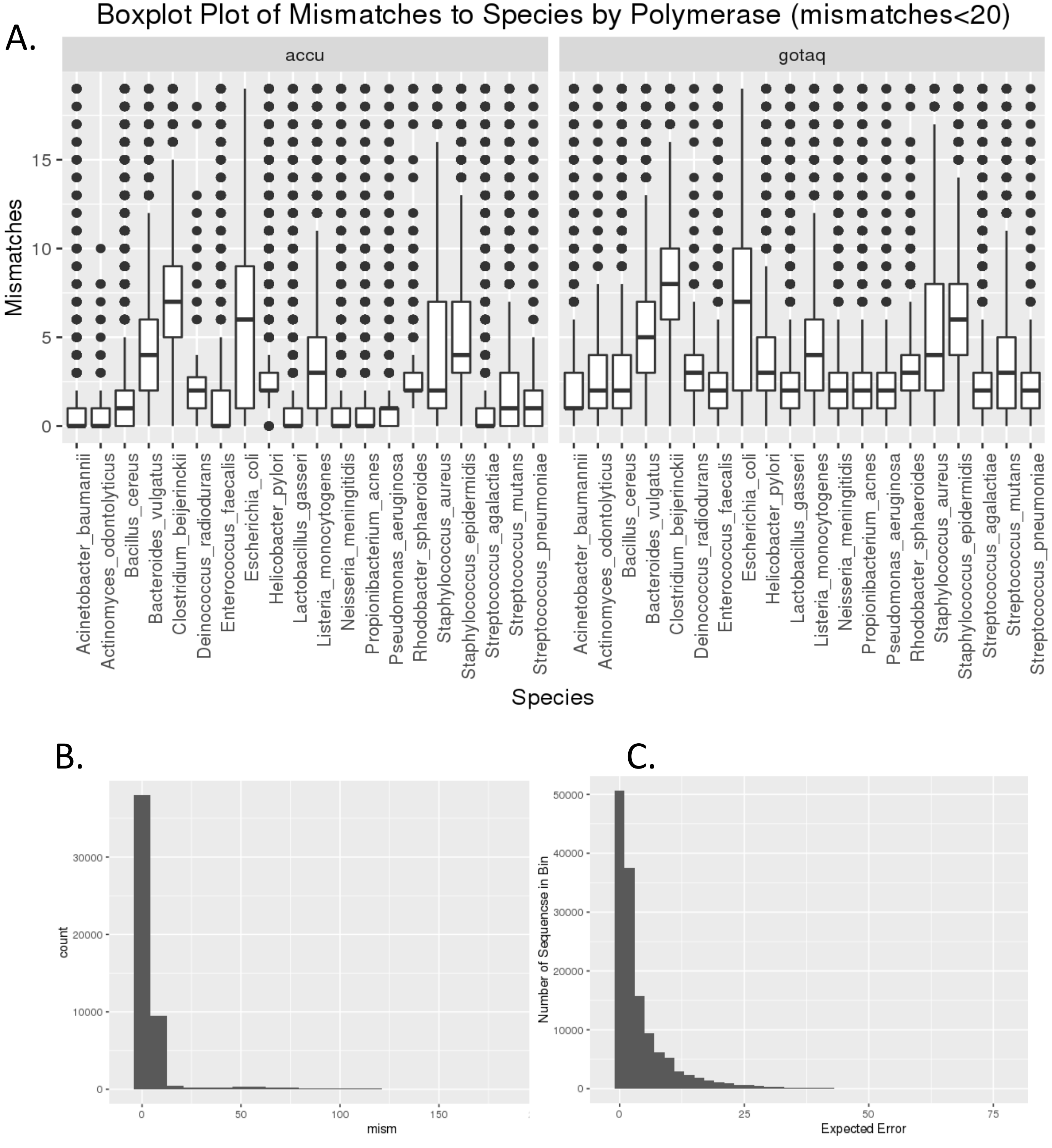

S13 Figure. *E.coli* MG1655 16S copies and MED analysis. A) Histograms showing the total number of errors between FL16S CCS reads collected from our lab stock of *E. coli* K12 MG1655 and the seven 16S rRNA genes in a whole genome assembly of the same stock. The reads were split into 6593 with EE>1 and 1445 with EE ≤ 1, as shown by the pink and blue lines, respectively. B) Maximum likelihood midpoint rooted tree from aligned MED representatives collected for CCS reads, along with *seven* 16S rRNA genes copies from closed Pacbio whole genome assembly of *E.coli*.

A.

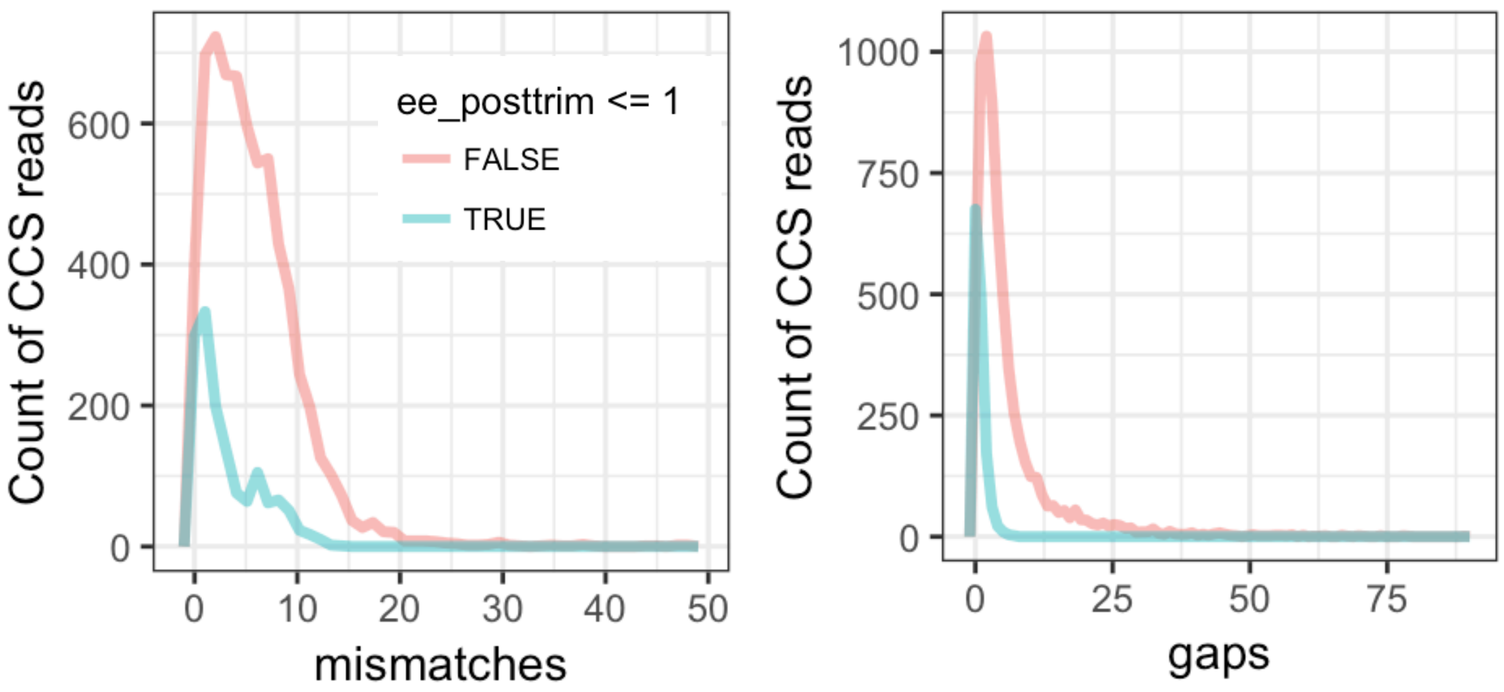

B.

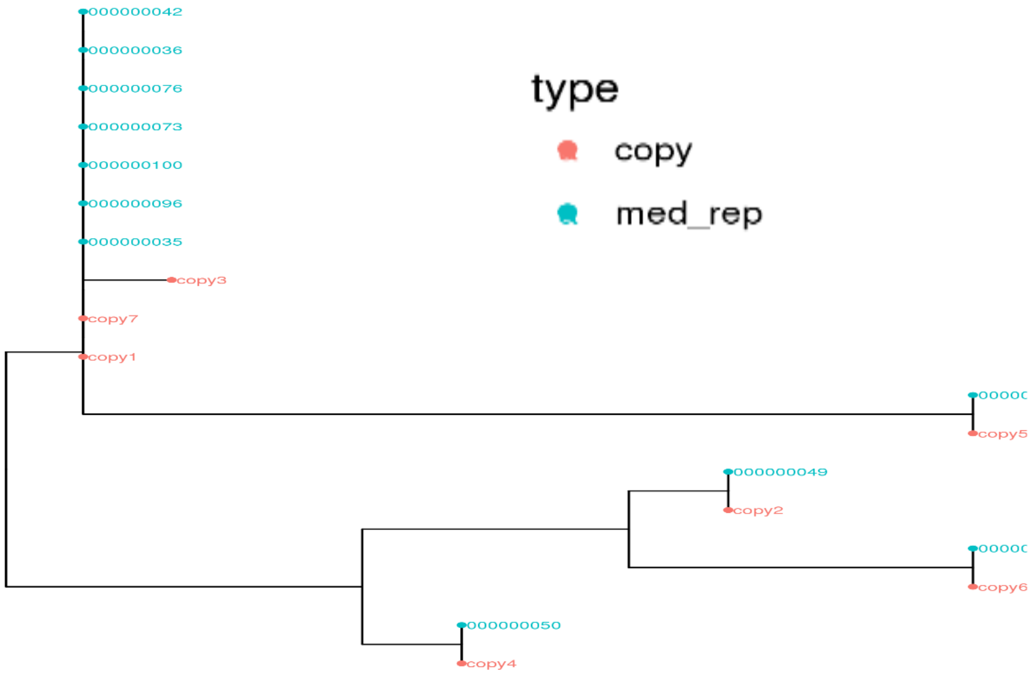

S14 Figure. Phylogenetic trees of well-resolved multi-species genera *Clostridium* and *Desulfovibrio*. Approximate ML trees were produced from all filtered EE≤1 CCS reads with or without truncation to the V3-V5 region that had been directly assigned to the select genus after multiple alignment. Tip colors represent the species labels belonging to the genus. Trees for *Clostridium* are shown (A) for FL16S and (B) for V3-V5. Trees for *Desulfovirbio* are show in (C) for FL16S and (D) for V3-V5.

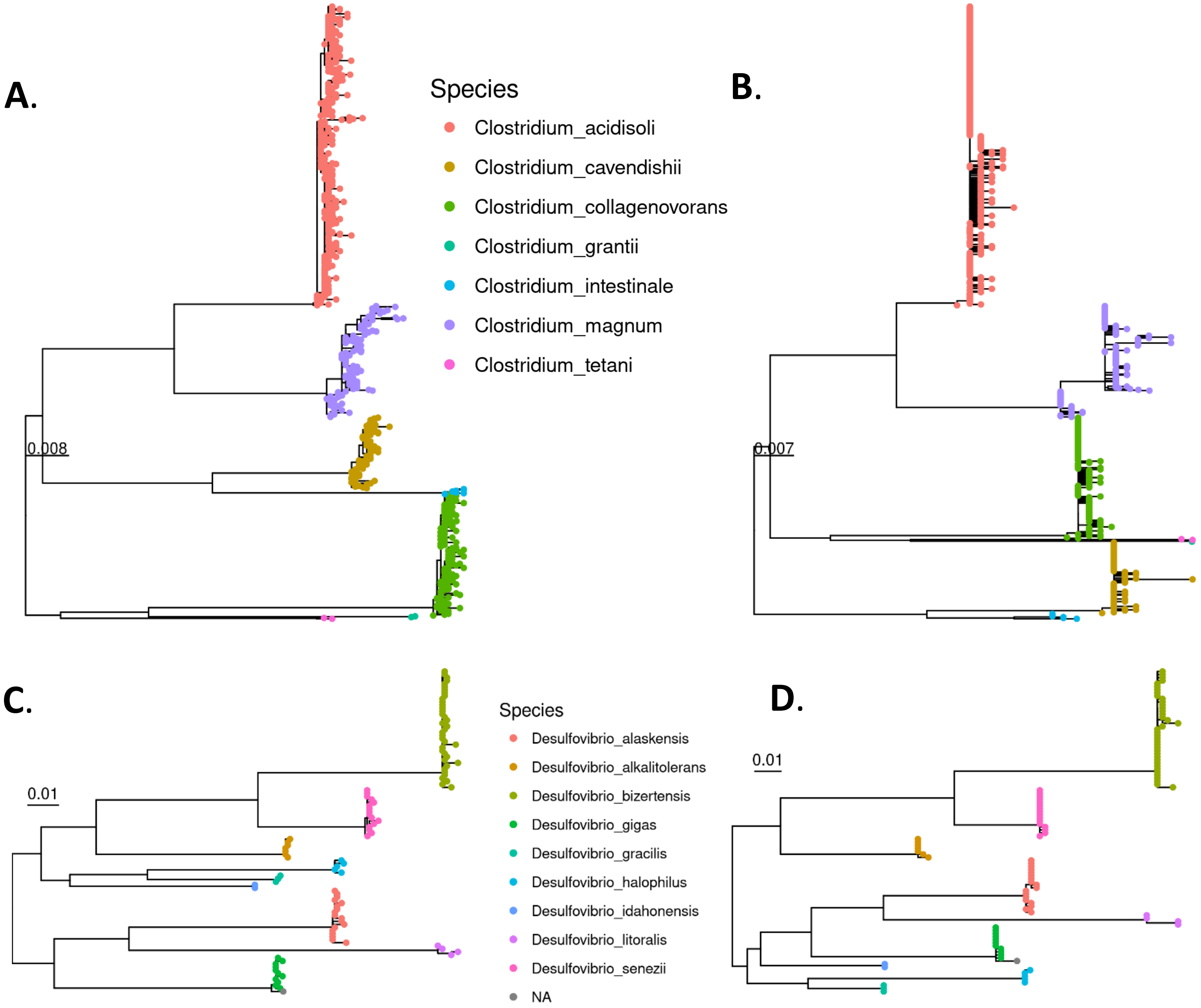

S15 Figure. Phylogenetic trees of poorly-resolved multi-species genera *Azotobacter* and *Nonlabens.* Approximate ML trees were produced from all filtered EE≤1 CCS reads with or without truncation to the V3-V5 region that had been directly assigned to the select genus after multiple alignment. Tip colors represent the species labels belonging to the genus. Trees for *Azotobacter* are shown (A) for FL16S and (B) for V3-V5. Trees for *Nonlabens* are show in (C) for FL16S and (D) for V3-V5.

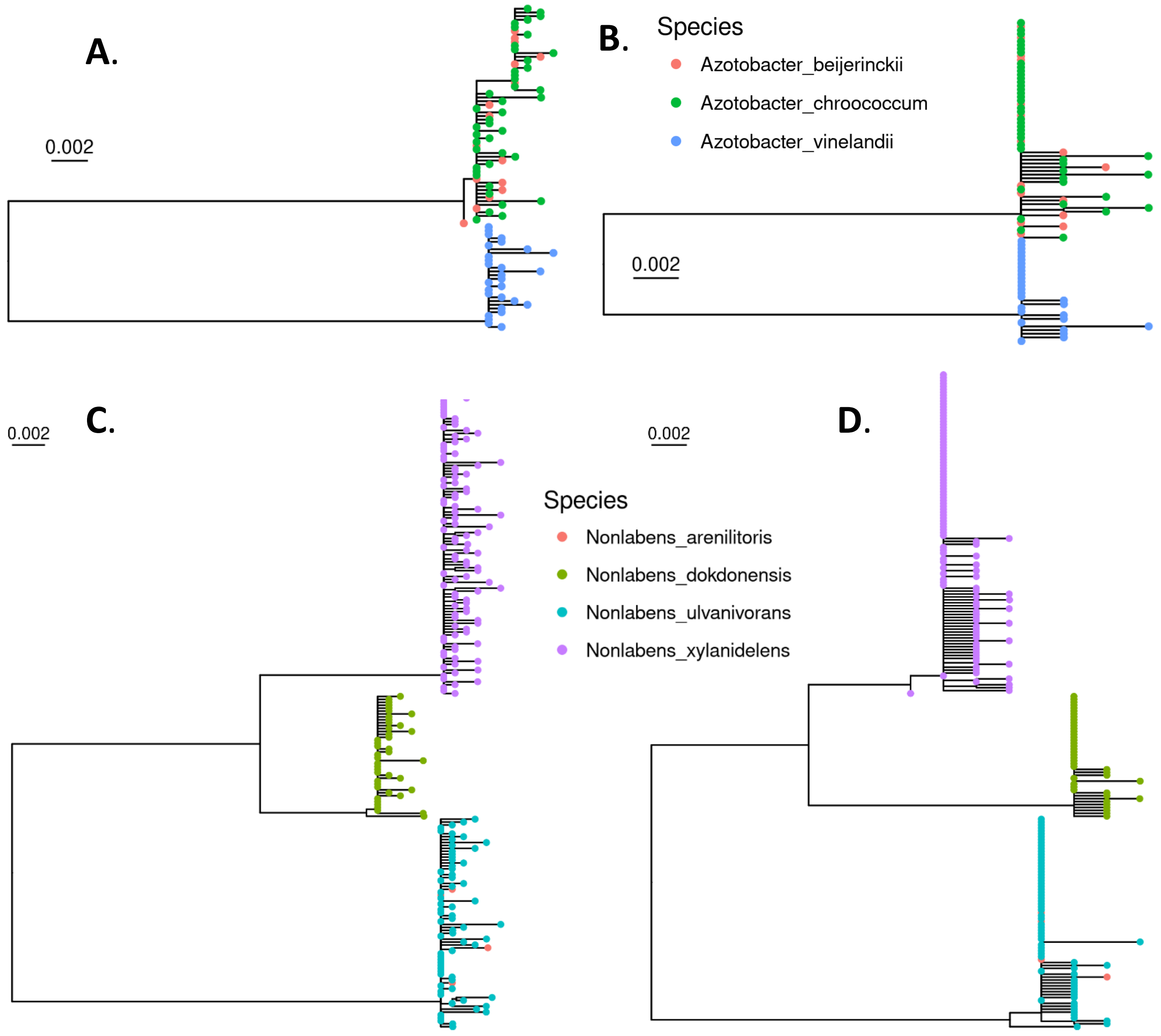

S16 Figure. Phylogenetic trees of multi-species genera with improved species resolution using FL16S for *Algoriphagus* and *Salegentibacter.* Approximate ML trees were produced from all filtered EE≤1 CCS reads with or without truncation to the V3-V5 region that had been directly assigned to the select genus after multiple alignment. Tip colors represent the species labels belonging to the genus. Trees for *Algoriphagus* are shown (A) for FL16S and (B) for V3-V5. Trees for *Salegentibacter* are show in (C) for FL16S and (D) for V3-V5.

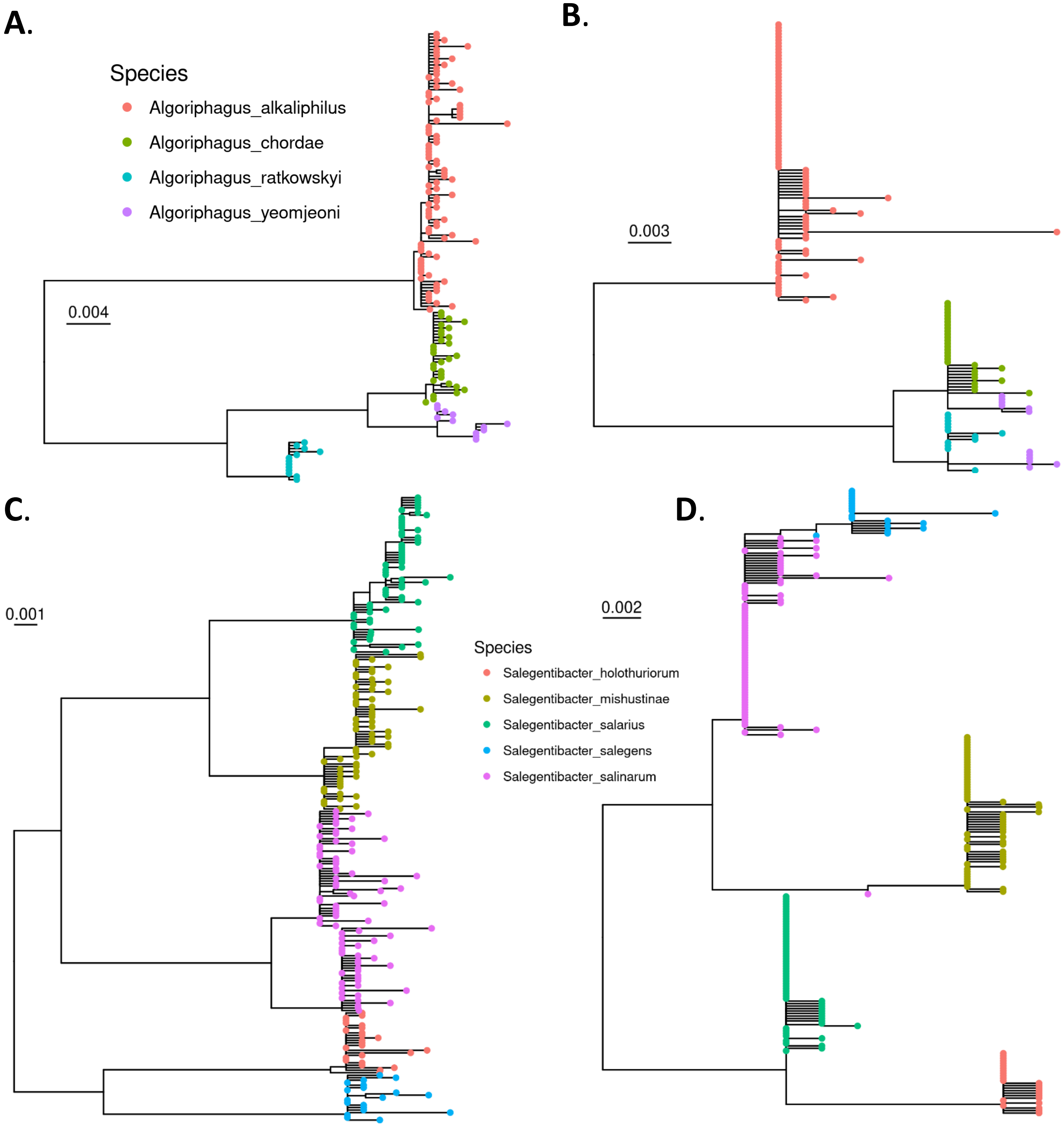

S17 Figure: Phylogeny of *Anaerococcus* MED nodes from the sinonasal communities plus NCBI database entries.

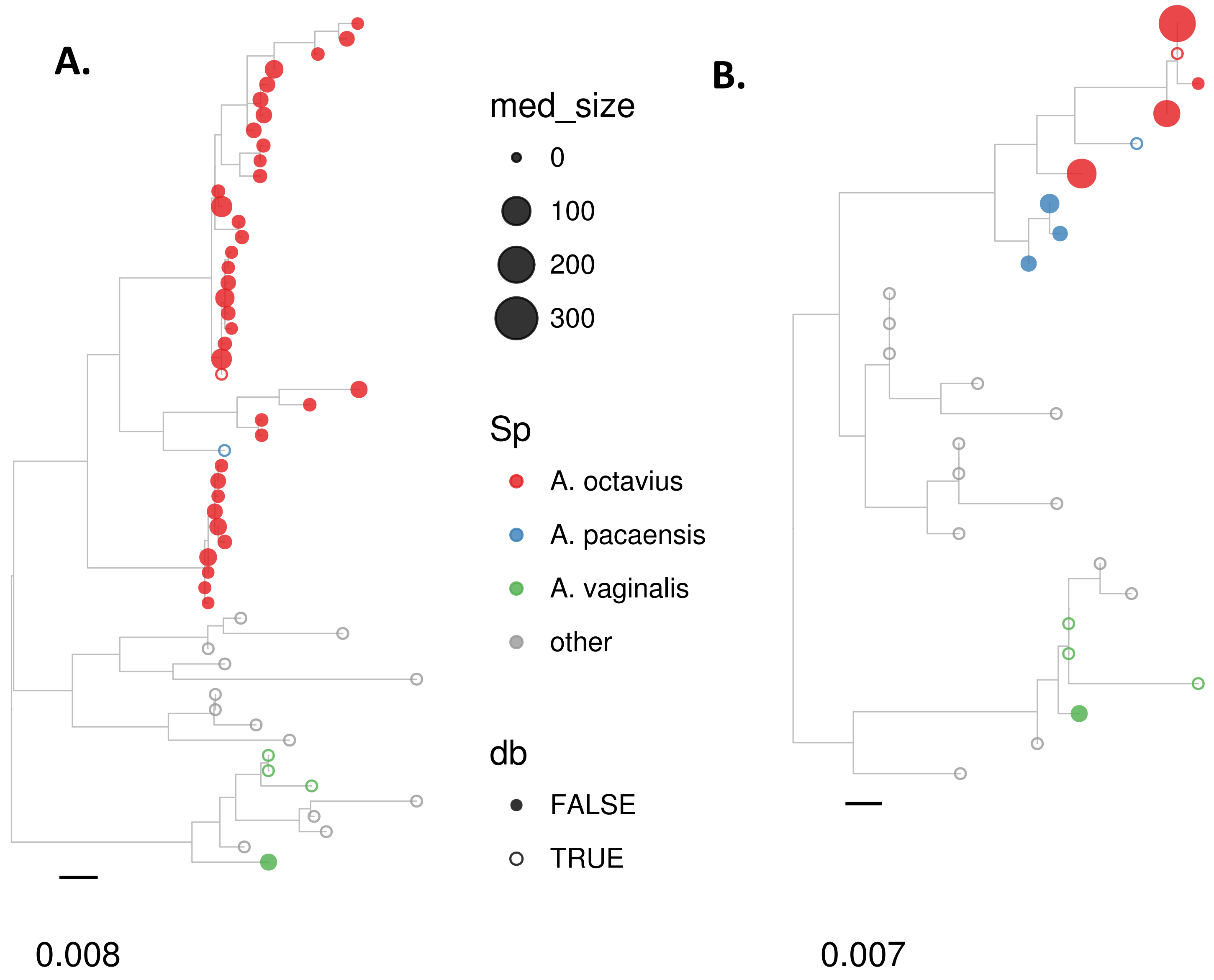

S18 Figure. Relationship between species-level confidence in centroid assignments and the number of species in the matching dbOTU. Relationship between species-level confidence in centroid assignments and the number of species represented in the matching dbOTU. Size of dot corresponds to the number of samples where that OTU was present. OTU in the lower left correspond to potentially novel taxa. Note all OTU at 42 dbOTU species on the x-axis correspond to Staphylococcus assignments.

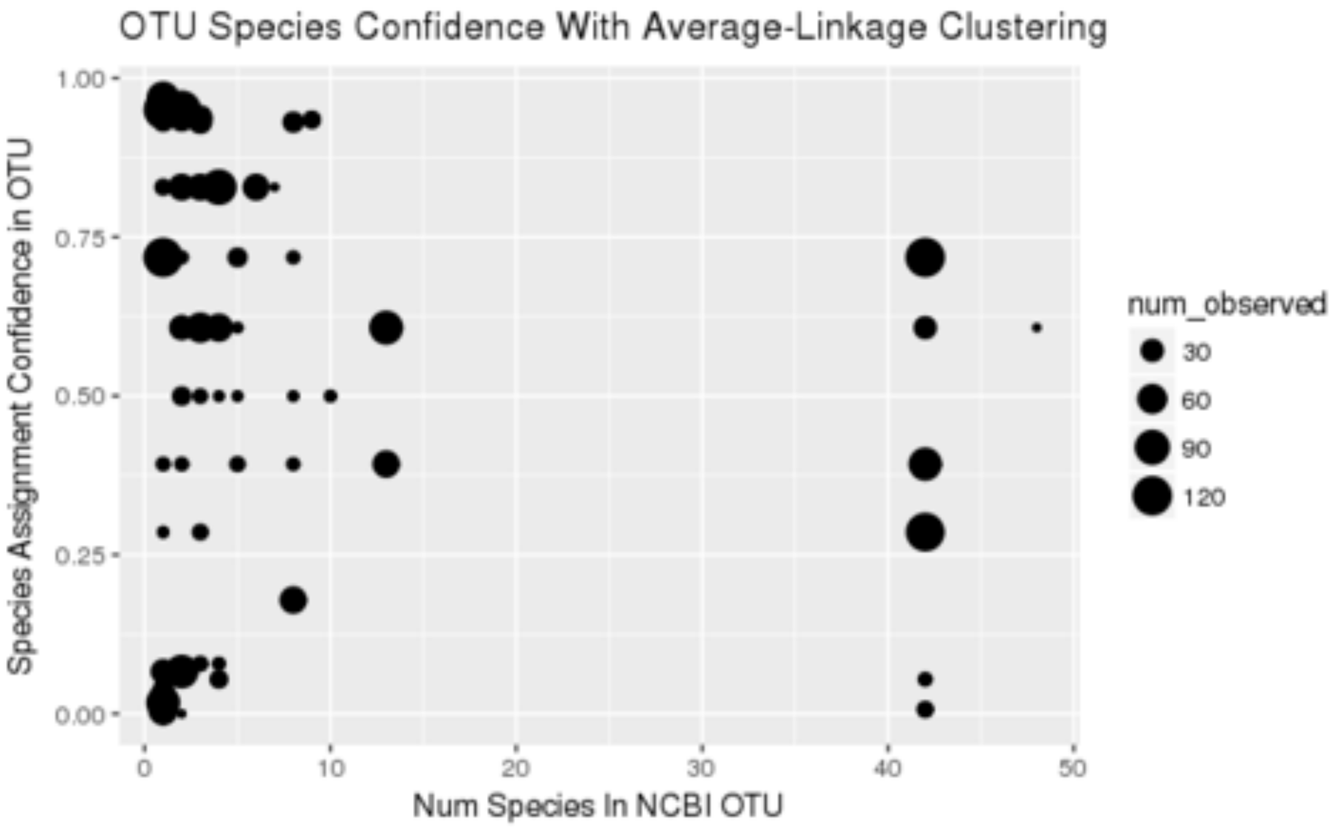

S19 Figure. Effective Number of Species as a function of sample read depth.

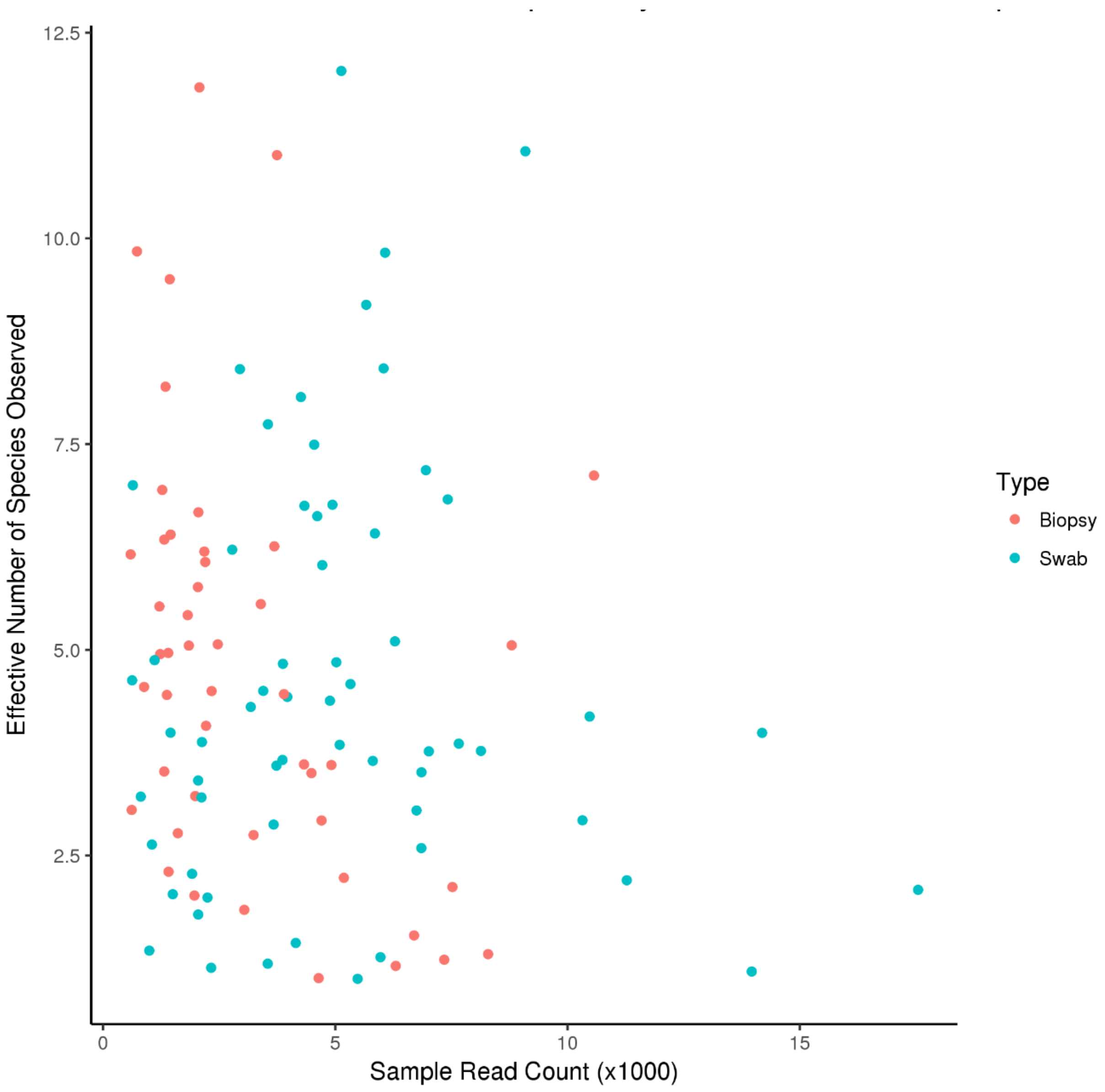

Supplement: Supplementary file 3 — Figure S1. Effect of primary filters on the number of reads. Figure S2. Insert size distribution. Figure S3. Effects of Host DNA on bacterial 16S yield. Figure S4. Total CCS yield vs. DNA yield vs. PCR yields. Figure S5. CCS reads mapping to the human genome from the sinonasal samples. Figure S6. Primer matching filters. Figure S7. Primer matching truncation and nucleotide variability against positive control E.coli forward and reverse primer matches. Figure S8. Primer matching truncation and nucleotide variability against positive control A.tumefaciens forward and reverse primer matches. Figure S9. Histogram of the number of species per DB cluster in NCBI. Figure S10. The effect of PCR cycle number and polymerase choice on OTU abundances in the BEI mock community. Figure S11. The effect of PCR cycles and polymerase on abundances of chimeric molecules in the BEI mock community. Figure S12. Substitution errors in BEI mock community. Figure S13. E.coli MG1655 16S copies and MED analysis. Figure S14. Phylogenetic trees of well---resolved multi---species genera Clostridium and Desulfovibrio. Figure S15. Phylogenetic trees of poorly---resolved multi---species genera Azotobacter and Nonlabens. Figure S16. Phylogenetic trees of multi---species genera with improved species resolution using FL16S for Algoriphagus and Salegentibacter. Figure S17. Phylogeny of AnaerococcusMED nodes from the sinonasal communities plus NCBI database entries. Figure S18. Relationship between species---level confidence in centroid assignments and the number of species in the matching dbOTU. Figure S19. Effective Number of Species as a function of sample read depth. (PDF 3167 kb) [file 40168_2018_569_MOESM3_ESM.pdf]
